# Roles of calcium signaling in cancer metastasis to bone

**DOI:** 10.37349/etat.2022.00094

**Published:** 2022-08-31

**Authors:** Tianying Xie, Sitong Chen, Jiang Hao, Pengfei Wu, Xuelian Gu, Haifeng Wei, Zhenxi Li, Jianru Xiao

**Affiliations:** 1 School of Health Science and Engineering, University of Shanghai for Science and Technology, Shanghai 200093, China; 2 Department of Orthopedic Oncology, Shanghai Changzheng Hospital, Shanghai 200003, China; 3 Center for Medical Genetics, School of Life Sciences, Central South University, Changsha 410008, Hunan, China; The University of Texas at Arlington, USA

**Keywords:** Bone metastasis, calcium, calcium channels, calcium-sensing receptor, calmodulin

## Abstract

Bone metastasis is a frequent complication for cancers and an important reason for the mortality in cancer patients. After surviving in bone, cancer cells can cause severe pain, life-threatening hypercalcemia, pathologic fractures, spinal cord compression, and even death. However, the underlying mechanisms of bone metastasis were not clear. The role of calcium (Ca^2+^) in cancer cell proliferation, migration, and invasion has been well established. Interestingly, emerging evidence indicates that Ca^2+^ signaling played a key role in bone metastasis, for it not only promotes cancer progression but also mediates osteoclasts and osteoblasts differentiation. Therefore, Ca^2+^ signaling has emerged as a novel therapeutical target for cancer bone metastasis treatments. Here, the role of Ca^2+^ channels and Ca^2+^-binding proteins including calmodulin and Ca^2+^-sensing receptor in bone metastasis, and the perspective of anti-cancer bone metastasis therapeutics via targeting the Ca^2+^ signaling pathway are summarized.

## Introduction

Bone metastasis is a process in which tumor cells escape from the primary tumor site and colonize the bone microenvironment [1], bringing about a plethora of complications, such as bone pain, pathological fractures, and life-threatening hypercalcemia. It has generally been characterized as osteolytic or osteoblastic (osteosclerotic), leading to bone destruction and new bone formation, respectively [2]. There’s a good reason for cancer cells’ predilection for bone. A seed-and-soil hypothesis was first proposed by Paget [3] in 1889, and shreds of evidence have been found over the years to support this hypothesis. Red marrow areas have a high blood flow, providing a nutritious environment [4]. More importantly, the bone microenvironment is rich in growth factors, including transforming growth factor β (TGFβ), insulin-like growth factor I (IGFI) and IGFII, fibroblast growth factors (FGFs), and calcium (Ca^2+^) [5]. These factors are released into the bone microenvironment and/or activated during bone resorption. Many of these growth factors can stimulate the proliferation of cancer cells in bone and induce the production and release of bone-resorbing factors from tumor cells [6]. For example, the secretion of receptor activator of nuclear factor-kappa B ligand (RANKL), located on the plasma membrane of osteoblasts, by activated T cells, binds the receptor activator of nuclear factor-kappa B (RANK) receptor on osteoclast precursors and leads to osteoclast formation [6]. Furthermore, tumor cells also secrete RANKL in a high Ca^2+^ environment and modulate osteoclastic differentiation [7]. The importance of RANKL in bone resorption and Ca^2+^ metabolism has been demonstrated clearly with the use of RANK knockout mice [8]. With findings in osteolytic metastatic lesions suggesting that the remolding of bone is induced by osteoclasts instead of tumor cells [2], RANK is determined to be essential for osteolytic metastases [9].

It is well established that Ca^2+^ signaling plays a pivotal role in tumor bone metastases with abundant research. Pathway enrichment analysis highlighted that the Ca^2+^ signaling pathway is a potential key regulator for breast cancer bone metastasis [10]. Elevated levels of intracellular Ca^2+^ in prostate cancer cells induce proliferation, angiogenesis, epithelial to mesenchymal transition (EMT), migration, and bone colonization [11]. Ca^2+^ signaling facilitates malignant cells’ bone colonization via a variety of mechanisms, interacting with cancer cells, osteoclasts, osteoblasts, and osteogenic niches [12]. However, the mechanism is not well understood. In this review, an insight into the Ca^2+^ signaling in cancer metastasis to bone is provided to our audiences.

## Ca^2+^

Ca^2+^, a ubiquitous intracellular messenger, regulates diverse cellular processes, such as gene transcription, apoptosis, autophagy, and cell proliferation. However, cellular Ca^2+^ signaling proteomes, such as Ca^2+^ channels, and Ca^2+^-binding proteins including calmodulin (CaM) and Ca^2+^-sensing receptor (CaSR), are tissue-specific and produce distinct Ca^2+^ signals suitable for tissue physiology [13]. Cytosolic Ca^2+^ signals practically participate in every aspect of cellular life, and rigorous regulation of Ca^2+^ homeostasis is important for preventing dysfunctions that lead to pathological changes [14]. In a pathological environment, remodeling of Ca^2+^ flux contributes to processes important for cancer progressions, such as uncontrolled proliferation, invasiveness of tumor cells, and the development of resistance to cancer therapies [15]. Increases in intracellular Ca^2+^ concentration are involved in cell migration, and impaired Ca^2+^ signaling is important in the metastatic behavior of tumor cells [16]. CaM1–4 remarkably emphasizes the importance of Ca^2+^ signaling by extending Ca^2+^ ions’ signals. Ca^2+^/CaM binding activates numerous proteins that contain CaM recruitment sites. Ca^2+^/CaM-dependent protein kinase IIs (CaMKIIs) are autophosphorylated and interphosphorylated after integrating with CaM, leading to prolonged kinase activity [17]. In addition to the elevated cytosolic Ca^2+^ concentration which contributes to major signaling function in most cells, extracellular Ca^2+^ is also an important physiological signal [18]. CaSR, an extracellular Ca^2+^ receptor, couples both various heterotrimeric G-proteins and downstream signaling pathways, mediating pluripotent effects [19].

## Ca^2+^ channels

The intricate fluxion of Ca^2+^ ions between extracellular and intracellular stores shapes the movement of Ca^2+^, such as Ca^2+^ release, Ca^2+^ oscillations, and Ca^2+^ spikes, modulating numerous biological functions [20, 21]. It is not surprising that the exchange of Ca^2+^ ions among different components of cells is interconnected and highly coordinated, and uncontrolled remolding of this well-connected network may lead to cancer cells metastasis to bone.

**Figure 1.**
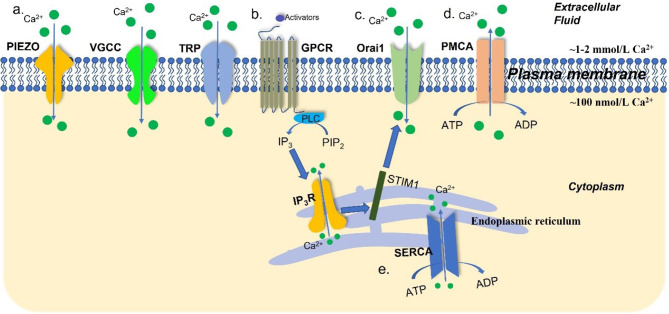
An overview of Ca^2+^ channels, transporters, and pumps in the plasma membrane and ER. Intracellular Ca^2+^ concentration is governed by a tightly mediated mechanism. (a) The TRP channels, VGCCs, and stretch-activated PIEZO channels are the Ca^2+^ channels and transporters in the plasma membrane; (b) after stimulation by activators, G-protein-coupled receptors (GPCRs) facilitate the dephosphorylation of phosphatidylinositol 4,5-bisphosphate (PIP_2_) into inositol 1,4,5-trisphosphate (IP_3_) by phospholipase C (PLC). In turn, IP_3_Rs initiate Ca^2+^ release from the ER; (c) STIM1 senses the depletion of the ER Ca^2+^ stores and activates Ca^2+^ influx via Orai1 Ca^2+^ channels; (d) PMCAs extrude Ca^2+^ ions from intracellular space to the extracellular space; (e) SERCAs transport Ca^2+^ from the cytoplasm into ER. ADP: adenosine diphosphate

Extracellular Ca^2+^ concentration is maintained at a high level (~1–2 mmol/L), which is 10–20,000 times that of the cytosolic Ca^2+^ concentration (~100 nmol/L). Endoplasmic reticulum (ER) stores intracellular Ca^2+^ ions, with a Ca^2+^ concentration around 100–400 μmol/L [22]. The regulation of this gradient is operated through a variety of mechanisms (Figure 1). Plasma membrane Ca^2+^ ATPases (PMCAs) and sarco(endo)plasmic reticular Ca^2+^ ATPases (SERCAs) are the main ATP-dependent channels that extrude Ca^2+^ ions from the cytosol to the extracellular space and ER, respectively. Inositol 1,4,5-trisphosphate receptors (IP_3_Rs) initiate Ca^2+^ releasing from the ER [23]. After the depletion of the intracellular Ca^2+^ stores, store-operated Ca^2+^ entry (SOCE), a specific Ca^2+^ influx pathway, initiates Ca^2+^ influx through Orai1 Ca^2+^ channels after activation by the ER Ca^2+^ store sensor stromal interaction molecule 1 (STIM1) [24, 25]. Extracellular Ca^2+^ ions enter the cytoplasm through substantial mechanisms and are the primary origin for intracellular Ca^2+^ signaling in cells. Examples include store-operated Ca^2+^ channels (SOCs), the transient receptor potential (TRP) superfamily of ion channels, voltage-gated Ca^2+^ channels (VGCCs) including L-, R-, N-, P/Q-, and T-type channels, and stretch-activated PIEZO channels [23, 25–28].

### TRP channels related to Ca^2+^

**Figure 2.**
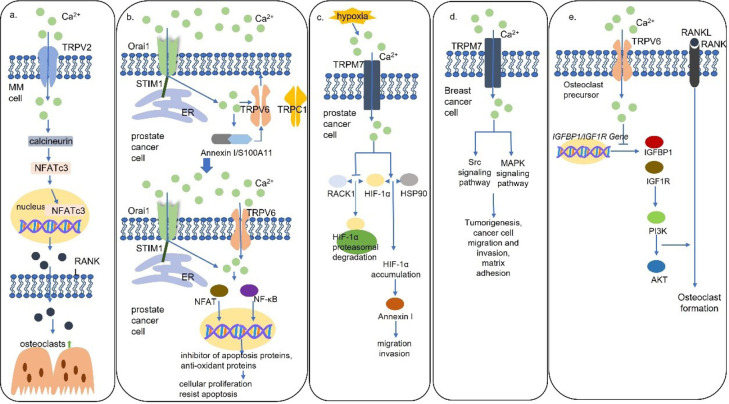
Schematic models for Ca^2+^ channels mediated Ca^2+^ signaling pathways. (a) TRPV2 mediates the secretion of RANKL via the Ca^2+^-calcineurin-NFATc3 signaling pathway in MM cells to activate osteoclast formation; (b) TRPV6 channels are translocated to the plasma membrane via the STIM1/Orai1 mediated Ca^2+^/Annexin I/S100A11 pathway. TRPV6 channels activate NFAT and nuclear factor κB (NF-κB) to promote proliferation, apoptosis resistance, and bone metastasis in prostate cancer cells; (c) hypoxia results in TRPM7-dependent hypoxia-inducible factor 1α (HIF-1α) accumulation which activates downstream Annexin I to promote cell migration and invasion in prostate cancer cells; (d) TRPM7 channels activate Src and mitogen-activated protein kinase (MAPK) signaling pathways to induce migration and invasion of breast cancer cells; (e) TRPV6 channels inhibit osteoclast formation by inhibiting the IGF/phosphatidylinositol 3-kinase (PI3K)/protein kinase B (AKT) signaling pathway. HSP90: heat shock protein 90; IGFBP1: insulin like growth factor binding protein 1; IGF1R: insulin like growth factor 1 receptor

The mammalian TRP cation channel superfamily has 28 family members [29]. While TRP melastatin 3α2 (TRPM3α2), TRP vanilloid 5 (TRPV5), and TRPV6 are highly Ca^2+^-selective, most TRP channels are nonselective [30]. Processes, such as cell apoptosis, proliferation, angiogenesis, invasion, and migration, are under the control of the regulation of the TRP cation channels in intracellular Ca^2+^ concentration (Table 1) [31]. Evidence has shown that TRPV2 mediates the secretion of RANKL via the Ca^2+^-calcineurin-nuclear factor of activated T cells 3 (NFATc3) signaling pathway in multiple myeloma (MM) cells, and RANKL levels are demonstrated in a Ca^2+^ dose-dependent way (Figure 2a) [7]. Furthermore, NFATc3 was found to bind to the promoter of RANKL and induce RANKL expression at the transcriptional level [7]. The RANKL-induced bone remolding contributed to the pathogenesis of MM lesions, but it also provided a likely treatment strategy.

**Table 1.** TRP channels and their functions in different cancers

**Family**	**Members**	**Cancer type**	**Effects**	**References**
TRPC	TRPC1	Colorectal cancer (CRC)	Enhanced cell proliferation, migration, invasion, and metastasis and apoptosis resistance	[32, 33]
	TRPC3	Gastric cancer	Tumorigenesis	[34]
		Breast cancer	Enhanced proliferation and apoptosis resistance	[35]
	TRPC5	CRC	Reduction in cancer differentiation	[36]
		Breast cancer	Chemotherapeutic resistance	[37]
	TRPC6	Hepatocellular carcinoma	Enhanced migration and invasion	[38]
		Breast cancer	Proliferation, migration, and invasion	[39]
		Oesophageal cancer	Essential for G2 phase progression	[40]
TRPV	TRPV2	Gastric cancer	Gastric cancer	[41]
	TRPV4	Gastric cancer	Enhanced proliferation, migration, and invasion	[42]
	TRPV6	Breast cancer	Tumor metastasis	[43]
TRPM	TRPM3	Clear cell renal cell carcinoma (RCC)	Tumor growth	[44]
	TRPM4	Prostate cancer	Enhanced proliferation	[45]
	TRPM7	Ovarian cancer	EMT and enhanced proliferation	[46, 47]

TRPC: TRP canonical

TRPV6 was found to be present at elevated levels in prostate, breast, thyroid, colon, and ovarian carcinomas [48]. Moreover, TRPV6 messenger RNA (mRNA) expression levels were upregulated with the malignant degree of prostate cancer and the highest levels of TRPV6 were detected in prostate cancer with lymphatic metastases and in recurrent lesions [49]. It was found that the TRPV6 channels are translocated to the plasma membrane involving the STIM1/Orai1/TRPC1-mediated ER Ca^2+^ store depletion via the Ca^2+^/Annexin I/S100A11 pathway, leading to increased proliferation and apoptosis resistance (Figure 2b) [50]. Furthermore, prostate cancer cells expressing TRPV6 were directly inoculated into the bone marrow cavity of tibias and promoted the generation of osteoblastic lesions suggesting TRPV6 promotes prostate cancer bone metastasis via numerous osteoblastic lesions [50]. It is well studied that the increased intracellular Ca^2+^ induced by TRPV6 dephosphorylates NFAT to induce cellular proliferation and regulates NF-κB oscillation to resist apoptosis, but the mechanism of the formation of the osteoblastic lesions is unknown [50].

The TRPM7 channels, which play a pivotal role in cell motility, are non-selective channels permeable predominantly to Mg^2+^ and Ca^2+^ [51]. In prostate cancer, an increase in serum Ca^2+^/Mg^2+^ ratio, which is regulated by TRPM7 and facilitates Ca^2+^ entry, leads to an increase in cell proliferation [52]. In a hypoxic environment, the increased TRPM7 results in HIF-1α accumulation. TRPM7-HIF-1α signaling activates downstream Annexin I protein expression mediating EMT, cell migration, and invasion (Figure 2c) [53]. In addition, it is also found that TRPM7 modulates the migration and invasion of breast cancer cells through the Src-MAPK signaling pathway (Figure 2d) [54]. Notably, TRPM7 overexpression promotes neuroblastoma cells to spread to the liver and bone marrow, but the mechanism is unknown [55].

It is well established that many members of the TRP cation channel superfamily play important roles in the mediation of tumor progression. The TRP cation channels also make a contribution to osteoclast differentiation, for example, TRPC1, TRPV4, and TRPV5 are essential for the regulation of osteoclastogenesis [56–58]. In a recent study, osteoporosis and enhanced bone absorption were found in TRPV6 knockout mice [59]. TRPV6 channels decreased the ratios of phosphorylation in the PI3K-AKT pathway which mediates the regulation of osteoclast formation and bone resorption (Figure 2e) [59]. The mechanism of the negative regulation of osteoclast differentiation and fusion and bone absorption by TRPV6 was revealed on a molecular level, and TPRV6 was confirmed to play an important role in bone metabolism [59]. Taken together with the formation of osteoblastic lesions induced by TRPV6, the TRPV6 channels play essential roles in the modulation of tumor progression and osteoclast activation. It makes one wonder, is there a connection between the TRPV6 generated osteoblastic lesions in prostate cancer and the TRPV6 negative regulated osteoclast differentiation? Further studies are required to elucidate the function of the TRP channels on cancer cell bone metastases and the possible link between metastases and the TRP regulation of osteoclast and osteoblast.

### SOCs

SOCE follows the depletion of ER Ca^2+^ storage. Both STIM1 and STIM2 are important for the maintenance of intracellular Ca^2+^ concentration [60]. After reduction of ER intraluminal Ca^2+^, STIM1 is activated and translocated to ER-plasma membrane junctions, where STIM proteins leash and gate Orai1 Ca^2+^ entry channels. STIM2 is more sensitive to changes in ER Ca^2+^ than STIM1, but it is a significantly weaker activator of Orai channels than STIM1.

In all, Orai1, -2, and -3 have been identified as plasma membrane Ca^2+^ channels. Although all three proteins are highly homologous to each other, they display notable differences in their features. Orai1 is the most potent to induce Ca^2+^ influx among its homologs, and its depletion significantly inhibits SOCE [61]. SOCE serves a wide set of signaling functions by elevating the cytosolic Ca^2+^ concentration. SOCE has potential roles in cellular proliferation and is inactivated during the division phase (M-phase) of the cell cycle. During the M-phase, STIM1 clustering is inhibited and Orai1 is internalized, thus uncoupling Ca^2+^ store depletion from Orai1 gating [62].

STIM1 and Orai1 are new targets for cancer treatment. Before STIM1’s role in Ca^2+^ influx was suspected, it was implicated that STIM1 could be a tumor suppressor [63]. The role of STIM and Orai in cancer is better studied particularly in the case of breast cancer. Breast cancer cell lines are not homogenous regarding STIM/Orai expression. Orai1 and STIM1 are predominant in the estrogen receptor-negative breast cancer cell lines, but Orai3 and STIM1/2 are the main SOCs in estrogen receptor-positive breast cancer cells [64]. The Orai3-induced Ca^2+^ influx contributed to breast cancer proliferation and survival but not in normal cells, consistent with the down-regulation of Orai3 arresting cell cycle progression and inducing apoptosis in breast cancer cells [65].

Focal adhesions, which are mediated by the interaction of integrin with the extracellular matrix, are relatively stable structures and tend to inhibit cell migration [66, 67]. Cell migration requires a dynamic state of focal adhesion [67]. STIM1 and Orai1 were shown to regulate tumor cell migration partially involving the mediation of the focal adhesion [68]. Increased Ca^2+^ influx might induce tumor cell migration depending on the activation of the focal adhesion kinase (FAK), the Ca^2+^-dependent protease calpain, and other Ca^2+^-sensitive proteins in focal adhesion turnover. SOCE inhibitor SKF96365 inhibited breast cancer cells’ metastasis in mouse models, providing a strong argument that SOCE is vital for breast tumor cell migration and metastasis.

Small conductance Ca^2+^-activated potassium channel protein 3 (SK3), a potassium channel, is a member of the small conductance Ca^2+^-activated potassium channel family [69]. An SK3-Orai1 complex, localized within lipid rafts, was found to be critical for the control of cancer cell migration and osteolytic bone metastases [70]. The SK3-Orai1 complex controls constitutive Ca^2+^ entry and tumor cell migration through store-independent Ca^2+^ signaling. Knocking down of the SK3 channels resulted in a lower metastatic score in breast cancer. Moreover, bone metastases achieved this lower metastatic score, but this reduction was not seen in lung metastases. The formation of the osteolytic lesions increased external Ca^2+^ concentration which amplified Ca^2+^ entry, establishing a vicious circle. Furthermore, the increased intracellular Ca^2+^ upregulated the activity of the Ca^2+^-sensitive protease calpain which could be attributed to bone metastases. Ohmline, a lipid inhibitor of SK3 channels [71], moved the SK3-Orai1 complex outside of lipid rafts and impaired the subsequent SK3-dependent Ca^2+^ entry, tumor cell migration, and bone metastases [70]. Therefore, ohmline could be a promising therapeutic application in preventing and treating breast cancer bone metastases. However, the role of calpain in breast cancer bone metastases needs further study.

Serum- and glucocorticoid-inducible kinase 1 (SGK1) mediates osteoclast differentiation, bone resorption, and bone metastasis via the Orai1 [72]. The expression levels of SGK1 are essentially upregulated during RANKL-induced osteoclastogenesis [72]. It was found that treatment with GSK650394, an SGK1 inhibitor, down-regulated Orai1 levels during osteoclastogenesis and overexpressed Orai1 markedly alleviated the inhibitory effects of GSK650394 on osteoclast differentiation [72]. In addition, SGK1 is functionally relevant for cell migration, which is critically dependent on SOCE [73], and treatment with GSK650394 significantly decreased breast cancer bone metastases in mouse models [72]. These findings present a new perspective on RANKL-induced osteoclastogenesis and breast tumor bone metastases through SGK1-mediated Orai1 overexpression. However, reintroduction of Orai1 did not fully rescue the GSK650394 abolished Ca^2+^ influx [72]. There are possibly other SGK1-mediated Ca^2+^ channels that can be regarded as future therapeutic targets.

### VGCCs

The VGCCs transport intracellular Ca^2+^ cations into intracellular Ca^2+^ transients initiating numerous physiological activities [26]. VGCCs are composed of three different subfamilies, the CaV1 (L-type) Ca^2+^ channel family, the CaV2 Ca^2+^ channel family, and the CaV3 (T-type) Ca^2+^ channel family, and are specified to ten members, CaV1.1, CaV1.2, CaV1.3, CaV1.4, CaV2.1, CaV2.2, CaV2.3, CaV3.1, CaV3.2, and CaV3.3 [74]. The CaV1 subfamily and the CaV2 subfamily are primarily responsible for the initiation of contraction, secretion, regulation of gene expression, integration of synaptic input in neurons, and synaptic transmission at ribbon synapses in specialized sensory cells, and initiation of synaptic transmission at fast synapses [26]. However, pieces of evidence have suggested that CaV3 mediates cellular processes including tumorigenesis and cancer progression by regulating intracellular Ca^2+^ levels [75].

T-type VGCCs expression levels are upregulated in many cancers and, thus, CaV3 channels are regarded as promising therapeutic targets. CaV3.1 isoform is a tumor-suppressor candidate and is reported to promote apoptosis and prevent tumor proliferation in breast cancer cells [76]. The CaV3.2 channels were not involved in the proliferation of MCF-7 breast cancer cells [76]. However, CaV3.1 was aberrantly upregulated and indicated a positive role in the regulation of proliferation in prostate cancer [77]. CaV3.1 together with CaV3.2 isoforms were found to increase gradually from normal skin to common nevi, dysplastic nevi, and melanoma samples with differences in distribution. Notably, metastatic melanoma showed the highest CaV3.2 expression levels which significantly differed from all other groups [78]. These results suggest that CaV3.1 and CaV3.2 channels may contribute to tumorigenesis and metastases. Therefore, further studies are required to identify the role of T-type VGCCs in cancers and bone metastasis, which is significant for the regulation of Ca^2+^ homeostasis.

### Connexin 43

**Figure 3.**
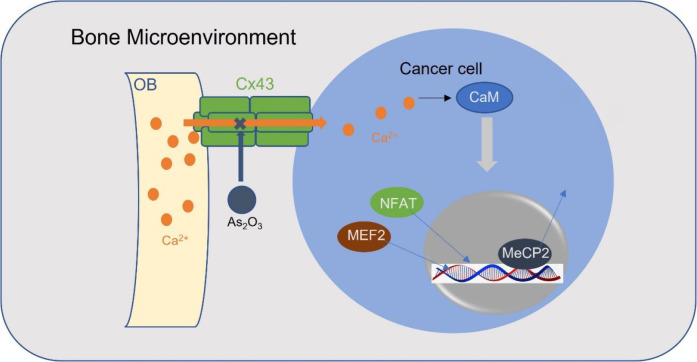
A schematic model for osteoblasts transports Ca^2+^ ions into cancer cells through Cx43 gap junctions. Ca^2+^ activates myocyte enhancer factor 2 (MEF2) and NFAT and releases methyl-CpG-binding protein 2 (MeCP2) from silenced promotors in a CaM-dependent manner. OB: osteoblast

Connexin 43 [Cx43, encoded by gap junction protein alpha 1 (*GJA1*)] belongs to the connexin family which is the major constituent of gap junctions, widely connects osteocytes and osteoblasts in bone, and directs Ca^2+^ flow [79, 80]. Prostate cancer and breast cancer bone metastasis showed the highest levels of Cx43 expression among all sites of metastases, suggesting that bone colonization requires Ca^2+^ flows from osteoblasts to cancer cells via the Cx43-based gap junctions [12]. Importantly, arsenic trioxide (As_2_O_3_) can inhibit Ca^2+^ signaling through downregulation of Cx43 and affecting Ca^2+^ influx, making it a promising therapeutic agent for clinical practice (Figure 3) [12].

## CaMs

Ca^2+^ transducer CaMs are prominent Ca^2+^ sensors [81]. Ca^2+^/CaM complexes bind to several classes of proteins and enzymes, including the CaM-dependent phosphatase calcineurin, myosin light-chain kinase, and Ca^2+^/CaMK family, as well as many other enzymes, channels, transport systems, and transcription factors (TFs) [82]. CaM-dependent proteins have been known in tumor progression, including cell migration, tumor cell invasiveness, and metastasis, and they are emerging as potential anti-cancer therapeutic targets. High expression levels of CaM were discovered in neuroblastoma tumor cells, and, especially, an abundance of CaM was seen in bone marrow metastases [83]. Treatment with tamoxifen, an anti-CaM drug, was effective against neuroblastoma with bone marrow metastasis in a dose-dependent manner, but not with liver metastasis.

### Ca^2+^/CaMKs

Ca^2+^/CaM-dependent protein kinase kinase α (CaMKKα) and β are the upstream kinases in the CaMK signaling cascade [84]. CaMKKα and β are activated through Ca^2+^/CaM binding and intramolecular phosphorylation. Activated CaMKKs phosphorylate and activate CaMKI and CaMKIV, adenosine monophosphate (AMP)-activated protein kinase (AMPK), or AKT (PKB). These kinases then phosphorylate downstream proteins, such as cyclic AMP (cAMP) response element-binding protein (CREB), activating transcription factor-1 (ATF-1), CAAT-enhancer-binding protein (C/EBP), and serum response factor (SRF) [85]. Notably, different from CaMKKα, which is solely dependent on Ca^2+^/CaM for activity, CaMKKβ also can be activated in the absence of Ca^2+^/CaM. Glycogen synthase kinase 3β (GSK3β) and cyclin-dependent kinase 5 (CDK5) regulate CaMKKβ activities through phosphorylation [86]. Unlike CaMKI and CaMKIV, CaMKII requires Ca^2+^/CaM complexes for activation, independent of CaMKKs [87].

CaMKKβ was identified as a downstream target protein of androgen receptor that has been known as prostate cancer bone metastasis enhancer (Table 2). Androgen-dependent regulation of CaMKKβ allowed tumor cells to migrate toward a more nutrient-rich environment, such as bone marrow, by activating and phosphorylating AMPK [88, 89]. Furthermore, CaMKKβ is a critical regulator of bone remodeling and macrophage function, creating a favorable microenvironment for colonizing and tumor growth of prostate cancer cells [90]. CaMKKβ stimulates osteoclast differentiation via CaMKKβ-CaMKIV-phosphorylated cAMP response element binding (pCREB) signaling cascade its downstream target, the NFATc1, the primary mediator during osteoclastogenesis [91]. However, in osteoblast, the CaMKKβ-CaMKIV pathway suppresses type I adenylate cyclase-cAMP regulated activities of protein kinase A (PKA), resulting in inhibited osteoblast differentiation [91]. Among immune cells, CaMKKβ was found to be restrictedly expressed in cells of the monocytic/macrophage lineage [92]. CaMKKβ ablation impaired macrophages’ ability such as cytokine secretion, and morphological changes, and CaMKKβ knockdown mice showed resistance to irritants that lead to systemic inflammation. Above all, dysregulation of CaMKKβ remodels bone into a favorable environment for tumor cells. Knockdown of CaMKKβ inhibits tumor growth, resists macrophage-induced inflammation, and improves the bone microenvironment. Further studies are still needed to investigate the molecular mechanisms of how CaMKKβ mediates prostate cancer cells’ metastatic abilities.

**Table 2.** Ca^2+^/CaMKs and their functions in different cancers

**Components**	**Cancer type**	**Effects**	**References**
CaMKK	Lung cancer	Tumor metastasis	[93]
	Prostate cancer	Tumor growth and castration resistance	[94]
	Glioma	Migration, invasion, and proliferation	[95]
CaMKI	Breast cancer	Control of cell cycle progression	[96]
CaMKII	Glioma	Migration and invasion	[97]
	Melanoma and hepatoma	Reprogramming of macrophages	[98]
	Prostate cancer	Inhibition of cancer growth and invasion, and induction of apoptosis	[99]
CaMKIV	Hepatic cancer	Cancer cell growth	[100]

Furthermore, increased cytolytic Ca^2+^ levels induced by Cx43 activate CaMKII mediating tumor cells’ bone colonization [12]. Nuclear Ca^2+^ signaling induces the CaMKII-dependent MeCP2 phosphorylation on serine 421 of MeCP2 and releases MeCP2 from silenced promotors in many cellular contexts [101, 102]. Decreased levels of MeCP2 enriched TFs, NFAT, and MEF2 which are associated with the promotion of EMT, migration, angiogenesis, and invasion [103, 104], in bone metastases. Moreover, evidence has suggested that CaMKII may be co-regulatory with the Notch signaling pathway which plays a critical role in the development of osteometric properties by prostate cancer bone metastatic cells [105].

It has been established that CaMKII is involved in the differentiation of both osteoblasts and osteoclasts. A collagen-binding motif derived from osteopontin induces an influx of extracellular Ca^2+^ via Ca^2+^ channels and promotes osteoblastic differentiation via Ca^2+^/CaMKII/extracellular signal-regulated kinase (ERK)/activating protein-1 (AP-1) signaling pathway [106]. More importantly, increased osteoclastic resorption and subsequent bone loss are common features of bone metastases. Once osteoclasts are stimulated, activated CaM complexes combine with CaMKII to regulate the expression of NFATc1 and tartrate-resistant acid phosphatase (TRAP/ACP5), an osteoclast marker, leading to macrophage differentiation into osteoclasts [107]. Zoledronic acid, a bisphosphonate, significantly decreases the Ca^2+^ levels, inhibits the expression of CaM and CaMKII, and prevents osteoclasts differentiation, providing effective therapy for patients with skeletal involvement from advanced cancers [107]. Furthermore, CaMKII induces *c-fos* gene expression and subsequent AP-1 activation, which can, in turn, drive NFAT2 expression and is involved in osteoclast differentiation and bone remodeling [108]. CaMKII also mediates leukemia inhibitory factor (LIF)-induced phosphorylation of serine-782 in the glycoprotein 130 (gp130) tail, which leads to internalization and downregulation of the gp130 receptor on the cell surface, suggesting that CaMKII may promote osteoclastogenesis by inhibiting the gp130 receptor signaling cascade [108]. Zoledronic acid has proven to be efficient to treat bone metastases targeting osteoclastogenesis, but zoledronic acid has nonnegligible side effects and limited application [107]. The molecular mechanisms of Ca^2+^ signaling leading to osteoclastogenesis may provide more specific targets to the treatment regimen for bone metastases, and needs further studies.

### Ca^2+^/CaM-dependent phosphatase

Calcineurin is a conserved Ca^2+^-CaM-dependent serine-threonine phosphatase that controls signaling pathways relevant to the migration, invasiveness, and metastatic potency of cancer cells. Increased cytolytic Ca^2+^ levels activate calcineurin mediating tumor cell bone colonization [12]. Calcineurin showed a similar effect as CaMKII to increase NFAT and MEF2 expression levels and inhibition of calcineurin also impedes bone colonization [12]. Calcineurin dephosphorylates resident NFAT proteins in the cytoplasm and triggers NFAT nuclear accumulation and activation [109]. RANK activation evokes Ca^2+^ oscillation by Ca^2+^ released from the ER and SOCE promotes CRC bone metastases through the calcineurin/NFATc1/ACP5 axis [110]. In addition, calcineurin/NFATc1 signaling promotes breast cancer metastasis to bone and brain and upregulates IGFI [111]. Regulator of calcineurin 1 isoform 4 (RCAN1.4) was found to reduce calcineurin activity and block nuclear translocation of NFATc1 [112]. Hence, RCAN1.4 is competent to reduce proliferation, migration, and metastases [112]. Moreover, RCAN1.4 was identified as a super suppressor of breast cancer and a potential therapeutic target for late-stage breast cancer patients with bone and brain lesions by ablation of calcineurin/NFATc1 signaling [111].

Furthermore, calcineurin Aα (CnAα), an isoform of calcineurin, is significantly overexpressed in small cell lung cancer (SCLC) tissues with bone metastasis in contrast to tumor cells where bone metastasis was absent [113]. CnAα is located in nuclear SCLC cells with bone metastases, but in non-metastatic tumors, CnAα is mainly located in the cytosol [113]. Downregulation of CnAα by lentiviral vector-mediated RNA interference (RNAi) reduced cell migration and invasion, and inhibited adhesion to the bone matrix, hampering metastasis development of SCLC with no change in the apoptosis rate of tumor cells [114].

## CaSR

CaSR is a GPCR that activates biased signaling in response to ligand stimulation [115]. With distinct ligand stimulation, CaSR preferentially activates relevant G proteins, including G_q/11_, G_i/o_, G_12/13_, and Gs, facilitating selective regulation of the wide array of cellular effects [115]. CaSR senses fluctuations in extracellular Ca^2+^ and regulates intracellular and extracellular Ca^2+^ concentrations [19].

It has been known that CaSR controls Ca^2+^ homeostasis through its modulation of the parathyroid glands and kidneys, therefore contributing to chondrocytes, osteoblasts, and osteoclasts differentiation, leading to skeletal development and bone turnover [116]. Moreover, the role of extracellular Ca^2+^ and CaSR in cancers has been identified, promoting tumor cell proliferation, migration, and bone metastasis [117]. Another study showed a high CaSR expression in RCC, and high extracellular Ca^2+^ levels enhanced migratory potential and proliferation of bone metastasizing primary RCC cells [118].

Parathyroid hormone-related protein (PTHrP) is important for the induction of osteoclasts maturation and differentiation. Unlike suppressed PTHrP secretion by elevated Ca^2+^ in normal tissue, high Ca^2+^ concentrations stimulate CaSR to secrete PTHrP in prostate cancer, breast cancer, and lung cancer cells [119–121]. These cancers are referred to as humoral hypercalcemia of malignancies (HHMs), because of their systemic secretion of PTHrP which induce the secretion of RANKL in osteoblast, which in turn promotes osteoclast formation [122]. This Ca^2+^-CaSR-PTHrP axis stimulates the differentiation of osteoclast precursors into mature osteoclast, therefore promoting bone resorption and Ca^2+^ release [122], initiating a vicious cycle, which contributes to the increased levels of Ca^2+^ and bone destruction.

In bone metastatic prostate cells, Ca^2+^/CaSR upregulates the expression of cyclin D1, a key component of the cell cycle, to support cancer cell growth, but this upregulation is absent in the nonskeletal metastases [123]. Furthermore, activation of CaSR triggered prostate cancer cells’ attachment, but the mechanism remains unknown [123]. In lung adenocarcinoma, CaSR was overexpressed in patients with bone metastasis, and overexpression of CaSR increased NF-κB protein levels and subsequent matrix metalloproteinases 2 and 9 to enhance tumor cell invasion [120]. These results suggested that CaSR facilitates the development of bone metastasis.

## Conclusions

The process and mechanism of bone metastasis are so complicated that there is no clear therapeutic target. The roles of Ca^2+^ signaling in tumor cells’ metastasis to the bone have been well established. As a ubiquitous second message, Ca^2+^ interacts with cancer cells to promote proliferation, migration, and invasion. Moreover, bone has the biggest Ca^2+^ storage in the human body and Ca^2+^ signaling mediates osteoclasts and osteoblasts differentiation which can facilitate bone metastasis. Thus, Ca^2+^ ions’ role in bone metastases is beyond tumor cells alone. It tells a better story along with osteoclasts, osteoblasts, and immune cells. Cancer cells’ colonization in the bone environment depends on the destroyed bone structures and systemic inflammation induced by immune cells. Increased concentrations of intracellular Ca^2+^ have been proven to contribute to the progress of bone metastasis. However, future scholars should also investigate whether Ca^2+^ acts as a negative regulator of bone metastases. Numerous Ca^2+^ channels and Ca^2+^ signaling pathways have provided us with a plethora of potential therapeutical targets for cancer treatment. However, many Ca^2+^-associated channels, proteins, and kinases have not been investigated, and for most of the signaling pathways that have been studied, the specific mechanisms in migration, invasion, and metastasis of different types of cancers are only just beginning to be understood. Medications targeting the Ca^2+^ signaling toolkit are limited. Therefore, a better understanding of the exact molecular functions and mechanisms of Ca^2+^ signaling in bone metastases is needed and further efforts can focus on the Ca^2+^ channels, Ca^2+^-related signaling cascades, and their effects on bone metastases.

## Abbreviations

AKT: protein kinase B
Ca^2+^: calcium
CaM: calmodulin
CaMKIIs: calcium/calmodulin-dependent protein kinase IIs
CaMKKα: calcium/calmodulin-dependent protein kinase kinase α
CaSR: calcium-sensing receptor
CnAα: calcineurin Aα
CRC: colorectal cancer
Cx43: connexin 43
EMT: epithelial to mesenchymal transition
ER: endoplasmic reticulum
gp130: glycoprotein 130
GPCR: G-protein-coupled receptor
HIF-1α: hypoxia-inducible factor 1α
IGFI: insulin-like growth factor I
IP_3_Rs: inositol 1,4,5-trisphosphate receptors
MAPK: mitogen-activated protein kinase
MeCP2: methyl-CpG-binding protein 2
MEF2: myocyte enhancer factor 2
MM: multiple myeloma
NFATc3: nuclear factor of activated T cells 3
NF-κB: nuclear factor κB
PI3K: phosphatidylinositol 3-kinase
PMCAs: plasma membrane calcium ATPases
PTHrP: parathyroid hormone-related protein
RANK: receptor activator of nuclear factor-kappa B
RANKL: receptor activator of nuclear factor-kappa B ligand
RCAN1.4: regulator of calcineurin 1 isoform 4
RCC: renal cell carcinoma
SCLC: small cell lung cancer
SERCAs: sarco(endo)plasmic reticular calcium ATPases
SGK1: serum- and glucocorticoid-inducible kinase 1
SK3: small conductance calcium-activated potassium channel protein 3
SOCE: store-operated calcium entry
SOCs: store-operated calcium channels
STIM1: stromal interaction molecule 1
TRP: transient receptor potential
TRPC: transient receptor potential canonical
TRPM3α2: transient receptor potential melastatin 3α2
TRPV5: transient receptor potential vanilloid 5
VGCCs: voltage-gated calcium channels

## Declarations

### Copyright

© The Author(s) 2022.
